# Community Health Centres as a model of care: contextualizing for Europe – a Delphi study

**DOI:** 10.1017/S1463423626101261

**Published:** 2026-06-16

**Authors:** Antonija Poplas Susic, Diederik Aarendonk, Jan De Maeseneer, Toni Dedeu

**Affiliations:** 1 Community Health Centre Ljubljana: Zdravstveni dom Ljubljana, Ljubljana, Slovenia; 2 Medical Faculty-Department for Family Medicine, University Ljubljana, Ljubljana, Slovenia; 3 European Forum for Primary Care (EFPC), Netherlands; 4 WHO collaborating centre Ghent, Belgium; 5 WHO Europe: World Health Organization, Regional Office for Europe, Denmark

**Keywords:** acceptability, Community Health Centres, Delphi study, Europe, feasibility, health policy, integrated care, Primary Health Care

## Abstract

**Aim:**

This study aimed to develop a European definition of Community Health Centres (CHCs) as a model of primary care, assess the acceptability and feasibility of this model among experts, and propose a common framework to guide CHC implementation across European countries.

**Background:**

CHCs have existed in Europe for more than five decades and are increasingly recognized as effective models for delivering comprehensive, equitable and integrated primary care. Despite their established global presence, there remains a need to contextualize and define CHCs within Europe’s diverse healthcare systems.

**Methods:**

Building on the International Federation of Community Health Centres (IFCHC) definition, a refined version comprising 35 key statements was developed through expert consultations. A two-round Delphi study was conducted with 31 experts from 16 countries, using five-point Likert-scale questionnaires to evaluate the acceptability and feasibility of each statement.

**Findings:**

All 35 statements were rated as acceptable and feasible (average value >3 on the five-point Likert scale). The most highly rated elements included respect for human rights, accessibility irrespective of socioeconomic status, interprofessional collaboration, and integrated, people-centred care. Although feasibility ratings were slightly lower than acceptability ratings, they improved in the second round, indicating increased consensus. Statements concerning governance, community participation, and responsibility for specific population subgroups received lower feasibility ratings. Overall, the findings demonstrate broad expert consensus on the relevance and adaptability of the CHC model within Europe. While some implementation challenges persist, particularly regarding governance, interprofessional collaboration, and intersectoral coordination, the proposed definition offers a robust foundation for strengthening primary care across diverse European contexts.

## Introduction

Primary care (PC) is widely regarded as the most crucial level of healthcare, capable of addressing more than 80% of the population’s health problems (Van Lerberghe *et al*., 2008). Building on foundational documents that position primary care as the cornerstone of health systems (Binagwaho *et al*., 2019; Kruk *et al*., 2018; Smith *et al*., 2017; Starfield *et al*., 2009; Starfield *et al*., 2010; Van Lerberghe *et al*., 2008; World Health Organisation, 2021; Toal-Sullivan *et al*., 2024; World Health Organisation, 2024), an appropriately integrated model of care could promote equity in treatment, ensure universal access, elevate health as a population priority, respond to evolving health needs, adhere to scientific recommendations, uphold quality standards, and enable GDPR-compliant analysis of anonymised health data. This model would also require professional adaptation and serve as a potential research platform (Brickley *et al*., 2021; Odone *et al*., 2016; Spoorenberg *et al*., 2018).

In recent decades, numerous factors have influenced primary care and the health of the population, such as population ageing, the rise of chronic illnesses, the introduction of palliative care, and the need to manage vulnerable patient groups. Patients have become more informed and demanding, regularly using modern digital tools, websites and ICT systems, which are increasingly integrated into clinical practices (Jane Osareme *et al*., 2024; Piera-Jiménez *et al*., 2024).

Alongside these new challenges, the organizational landscape of primary care varies widely across European countries, encompassing individual practices, group practices, cooperative practices and CHCs. Differences in primary health care (PHC) settings, financing mechanisms, organizational structures and available resources create a pressing need for a shared framework to ensure equitable access to primary care across Europe.

Over 45 years since the Alma-Ata Declaration (International Conference on PHC, 1978; Chan *et al*., 2008), awareness of CHCs and their integrated approach to healthcare and wellbeing continues to grow, with increasing focus on supporting patients’ life goals (De Maeseneer *et al*., 2012; Kluge *et al*., 2019). CHCs are emerging as an influential model for operationalizing global visions and commitments to PHC by governments and non-governmental organizations (De Maeseneer *et al*., 2019).

CHCs, as a model of care, have long existed as robust institutions delivering PHC throughout Europe. Decades ago, they were described as functional organizations established within communities to serve both individuals and populations (Stampar *et al*., 1955).

According to the International Federation of Community Health Centres (IFCHC, https://www.ifchc.org/), CHCs are defined by the following core attributes (International Federation of CHCs, 2013), which are widely recognized:

- Delivery of comprehensive PHC
- Responsibility for a defined population
- Integration of broader determinants of health into routine clinical practice
- A strong commitment to equity and social inclusion
- Emphasis on community engagement and civic participation
- Contribution to universal health coverage, with a firm commitment to accessibility

In Europe, the European Forum for Primary Care (EFPC), alongside its Community Health Centre Working Group (WG CHC), has examined whether this core definition remains conceptually appropriate and temporally relevant for the EU context, and whether further refinement or expansion is warranted. This effort seeks to develop a care model responsive to contemporary population needs and supportive of healthcare professionals working collaboratively with informal caregivers and local communities. Crucially, the initiative respects the autonomy of national health systems and does not seek to interfere with jurisdictional authority.

**The objectives of this paper are to:**

- Develop a definition that conceptualizes CHCs as a model of care in the European context
- Assess the acceptability and feasibility of the proposed definition
- Based on the findings, propose a common template for implementing CHCs as a model of care across Europe

## Method

### Community Health Centre’s definition

The definition of CHCs developed by the International Federation of Community Health Centres (IFCHC) served as the foundation for formulating a Europe-specific definition and assessing its acceptability and feasibility. This initial definition was first discussed within the WG CHC and subsequently divided into 27 individual statements to facilitate further analysis, each capturing a key component of the original definition (see Table 1). These statements were presented at a EFPC workshop held prior to the COVID-19 pandemic, where participants were invited to provide feedback and propose refinements.

**Table 1.** The final definition of the Community Health Centres (IFCHC: statements 1-27; added statements: 28–35) Table 1 long description.

1	Community Health Centre is defined as a model of care in the specific region/ municipality/ district/ geographic area.
2	Community Health Centres deliver integrated, comprehensive and people-centred primary health care.
3	Community Health Centres deliver a care model through an interprofessional team.
4	Community Health Centres address aspects of both health and wellbeing.
5	Clinical primary care services at Community Health Centres address aspects of health promotion and illness prevention.
6	Clinical primary care services at Community Health Centres address aspects of curative care and rehabilitation.
7	Clinical primary care services at Community Health Centres use a holistic frame of reference.
8	Clinical primary care services at Community Health Centres are orientated towards the needs of individuals.
9	Clinical primary care services at Community Health Centres are orientated towards the needs of families.
10	Clinical primary care services at Community Health Centres are orientated towards the needs of populations.
11	Clinical primary care services at Community Health Centres are orientated towards the needs of communities.
12	The Community Health Centre care team integrates into its daily activities, attention to the broader causes of illness.
13	The Community Health Centre care team looks at the social determinants of health.
14	The Community Health Centre care team addresses social determinants of health through intersectoral cooperation.
15	Community Health Centres develop a community-oriented primary care strategy.
16	Community Health Centres blend skills for individual health care with approaches focused on public health.
17	Community Health Centres have a commitment to equity and social inclusion.
18	Community Health Centres put emphasis on access to health care (with special attention given to the most vulnerable).
19	Community Health Centres respect fundamental human rights.
20	Community Health Centres place a strong emphasis on community engagement and civic participation in health and health care.
21	Community Health Centres may include participation of clients/ patients and other community members in governance of the healthcare organisation.
22	Community Health Centres contribute to universal coverage.
23	Community Health Centres are strongly committed to being accessible for individuals and families, irrespective of race, religion, social status and other factors, including ability to pay for care.
24	Community Health Centres engage in processes of continuous quality improvement.
25	Community Health Centres engage in needs of individuals and patients that they are serving.
26	Community Health Centres take responsibility for a population that is geographically-determined.
27	Community Health Centres take responsibility for a population that is defined by population group(s).
28	Collaboration between care providers in the Community Health Centre is important.
29	Collaboration between Community Health Centres (CHCs) (CHC = a model of care) is important.
30	Collaboration between different region that provide health care is important.
31	Spreading and supporting good practices in/ between CHCs is important.
32	Spreading and supporting good practices in the country/ between countries are important.
33	Coordination the care providers in the Community Health Centre is important.
34	Coordination between Community Health Centres (CHCs) (CHC = a model of care) is important.
35	Coordination between different region that provide health care is important.

Subsequent discussions on whether the IFCHC definition within 27 statements, adequately reflects the concept of CHCs as an organizing model of care, or whether adaptation is required, were conducted with primary care professionals from various European countries during workshops held at international congresses, including the European Association for Quality and Safety in General Practice/Family Medicine (EQuiP) Conference, the Association of General Practice/Family Medicine of South-East Europe (AGP/FM SEE) Conference, the World Organization of Family Doctors (WONCA) Congress, and the European Center for Peace and Development (ECPD) Congress. These workshops were facilitated by WG CHC members, who manually recorded any new aspects or elements not captured in the original 27 IFCHC statements. Participants, primarily primary healthcare professionals and numbering from fewer than 10 to more than 30 per session, contributed insights aimed at shaping a model of care that meets the needs of both patients and care providers in the current context. Any new content emerging from these discussions was first reviewed within the WG CHC and subsequently prepared for further deliberation at the next workshop.

Based on feedback from these consultations, the 27 statements of the IFCHC definition were expanded to a total of 35. The additional eight statements (statements 28 to 35 in Table 1) addressed cooperation, networking, and the dissemination of good practices among and between CHCs. The revised definition, comprising all 35 statements, was subsequently reviewed in collaboration with representatives of the World Health Organisation (WHO) working with the EFPC. Finally, the complete version was formally discussed and confirmed once again during an EFPC workshop meeting. These 35 statements were then prepared for assessment regarding their acceptability and feasibility across EU countries.

### Questionnaire

All 35 statements were incorporated into a Delphi study questionnaire. A five-point Likert scale was used to assess agreement with each statement (1 = strongly disagree, 2 = disagree, 3 = neither agree nor disagree, 4 = agree, 5 = strongly agree). Two parallel versions of the questionnaire were developed: one assessing the acceptability of each statement, and the other evaluating the feasibility of implementing each statement in practice.

Participants were also asked to provide general information about their institution or employing organization, area of work, profession and region (rural or urban). The questionnaire did not allow for additional comments.

For analysis, an average score of 3 or below for any statement was classified as disagreement, while an average score above 3 was considered agreement.

### Participants

The EFPC secretariat compiled a purposive sample of 62 individuals with in-depth knowledge of primary care systems, representing diverse professional backgrounds, including group practices,CHCs, professional organizations, academic institutions and state agencies. The candidate list included each individual’s name, country, institution or organization, area of work, profession, email contact and region (rural or urban). A unique questionnaire code was assigned to each participant to ensure anonymity. Experts from 25 countries, including both EFPC member and non-member states, were invited to participate. In total, 31 experts from 16 countries responded, yielding a 50% response rate. All participants who completed the first round also completed the second round.

### Delphi study

A Delphy study was conducted to evaluate the level of agreement regarding the acceptability and feasibility of the 35 proposed statements. The Delphi method was selected because it enables anonymous participation across multiple countries, allows respondents to reconsider their views in light of group feedback and facilitates the inclusion of geographically diverse experts in a cost-effective manner. The absence of direct interaction among participants also minimized the influence of dominant individuals and supported the development of an unbiased expert consensus.

Questionnaires were distributed via email, and participants were asked to rate each statement using a 5-point Likert scale. No strict time limit was imposed, as the objective was to obtain responses from all invited participants; therefore, several follow-up reminders were issued individually.

In the first round, participants indicated their level of agreement with each statement. After all responses were collected, the average score for each statement was calculated.

In the second round, participants received the same set of statements, now accompanied by the average scores from the first round. They were invited to reconsider their initial assessments in light of the group’s aggregated feedback and to indicate whether their views had changed. The final average values from the second round represented the overall level of consensus regarding the acceptability and feasibility of the proposed CHC definition.

### Statistical analysis

Descriptive statistics (mean, standard deviation, median and minimum/maximum values) were calculated for each statement using Excel. No comparative analyses were conducted between different work settings, rural and urban regions or Western and Eastern European countries, as the aim of the study was to develop a uniform template for CHCs as a model of care across Europe.

Ethical approval: Ethical approval was not required for this study, as it did not involve patients or the collection of personal health data.

## Results

### Participants

Of the 62 individuals invited to participate, 31 experts responded over a seven month period, yielding a response rate of 50%. These participants represented 16 European countries (see Table 2).

**Table 2.** Participants Table 2 long description.

Country	Number	Percent
Belgium	3	9,7
Bosnia and Herzegovina	1	3,2
Croatia	2	6,5
Denmark	1	3,2
Estonia	1	3,2
France	1	3,2
Germany	3	9,7
Italy	2	6,5
Macedonia	1	3,2
Malta	2	6,5
Montenegro	1	3,2
Netherlands	3	9,7
Portugal	2	6,5
Slovenia	3	9,7
Spain	2	6,5
Turkey	3	9,7
**Total**	**31**	**100,0**

In terms of geographical distribution, 25 participants reported working primarily in urban settings, while 6 were based in rural areas. Participants were drawn from a range of professional environments, with almost 42% working within CHCs (see Table 3).

**Table 3.** Working settings Table 3 long description.

Working IN	N	Percent
Group practice	3	9,7
Community Health Centre	13	41,9
Others (e.g. university)	5	16,1
Professional organisation	4	12,9
State institution	6	19,4
**Total**	**31**	**100,0**

Some respondents did not complete all general information fields in the questionnaire; in several cases, professional background data were missing, although information on institutional affiliation or field of work was provided.

### A Delphi study

General participant data were not fully completed by all respondents; however, no missing values were observed for the statement ratings.

The study extended over a relatively long period. Due to limited responsiveness in the first round, the study coordinator issued repeated reminders. After seven months, the first round was completed with 31 respondents out of 62 invited experts. Only those who participated in the first round received the second-round questionnaire, which included the calculated mean scores from round one. The second round also required several months to complete, but after multiple follow-up reminders, all first-round participants submitted their responses. No data loss occurred, as all statements were rated in both rounds.

Following the second round, consensus was achieved on all 35 statements, each reaching a mean score of ≥3. The lowest mean score for acceptability was 3.61, and for feasibility 3.52, with the exception of the statement *Community Health Centres may include participation of clients/patients and other community members in governance of the healthcare organisation*, which received a feasibility score of 3.29. Table 4 presents a comparison of the first-round results (individual agreement with each statement) and the second-round results (consensus among participants) for both acceptability and feasibility of the CHC model.

**Table 4.** Delphy study. Results of the first round (agreement with the statements) and the second round (agreement with each other) related to the acceptability and feasibility of the definition Table 4 long description.

No.	Definition of the CHC’s as a model of care	Agreement with the acceptability of the statement		Agreement with the feasibility of the statement	
	Statements (IFCHC statements 1-27; EFPC WG statements 28-35)	1st round	2nd round	1st round	2nd round
1	Community Health Centre is defined as a model of care in the specific region/ municipality/ district/geographic area.	4,31	4,58	3,66	4,23
2	Community Health Centres deliver integrated, comprehensive and people-centred primary health care.	4,56	4,74	3,88	4,19
3	Community Health Centres deliver a care model through an interprofessional team.	4,44	4,61	3,72	4,23
4	Community Health Centres address aspects of both health and wellbeing.	4,31	4,52	3,81	4,03
5	Clinical primary care services at Community Health Centres address aspects of health promotion and illness prevention.	4,47	4,65	4,03	4,35
6	Clinical primary care services at Community Health Centres address aspects of curative care and rehabilitation.	4,28	4,29	4,06	4,13
7	Clinical primary care services at Community Health Centres use a holistic frame of reference.	4,13	4,35	3,66	3,94
8	Clinical primary care services at Community Health Centres are orientated towards the needs of individuals.	4,44	4,35	4,09	4,29
9	Clinical primary care services at Community Health Centres are orientated towards the needs of families.	4,00	3,90	3,59	3,68
10	Clinical primary care services at Community Health Centres are orientated towards the needs of populations.	3,91	4,10	3,53	3,77
11	Clinical primary care services at Community Health Centres are orientated towards the needs of communities.	4,03	4,32	3,47	3,74
12	The Community Health Centre care team integrates into its daily activities, attention to the broader causes of illness.	4,31	4,32	3,56	3,71
13	The Community Health Centre care team looks at the social determinants of health.	4,41	4,52	3,56	3,94
14	The Community Health Centre care team addresses social determinants of health through intersectoral cooperation.	4,19	4,35	3,38	3,74
15	Community Health Centres develop a community-oriented primary care strategy.	4,31	4,45	3,38	3,74
16	Community Health Centres blend skills for individual health care with approaches focused on public health.	4,25	4,42	3,59	3,71
17	Community Health Centres have a commitment to equity and social inclusion.	4,47	4,68	3,75	3,77
18	Community Health Centres put emphasis on access to health care (with special attention given to the most vulnerable).	4,38	4,58	3,94	4,16
19	Community Health Centres respect fundamental human rights.	4,50	4,84	4,28	4,42
20	Community Health Centres place a strong emphasis on community engagement and civic participation in health and health care.	4,00	4,19	3,28	3,68
21	Community Health Centres may include participation of clients/ patients and other community members in governance of the healthcare organisation.	3,78	3,71	3,22	3,29
22	Community Health Centres contribute to universal coverage.	4,34	4,61	3,97	4,19
23	Community Health Centres are strongly committed to being accessible for individuals and families, irrespective of race, religion, social status and other factors, including ability to pay for care.	4,66	4,84	4,28	4,48
24	Community Health Centres engage in processes of continuous quality improvement.	4,47	4,68	3,97	4,16
25	Community Health Centres engage in needs of individuals and patients that they are serving.	4,38	4,61	3,91	4,13
26	Community Health Centres take responsibility for a population that is geographically-determined.	4,28	4,35	3,78	3,90
27	Community Health Centres take responsibility for a population that is defined by population group(s).	3,53	3,61	3,34	3,61
28	Collaboration between care providers in the Community Health Centre is important.	4,56	4,77	3,84	4,10
29	Collaboration between Community Health Centres (CHCs) (CHC = a model of care) is important.	4,22	4,42	3,72	3,84
30	Collaboration between different region that provide health care is important.	4,16	4,26	3,41	3,65
31	Spreading and supporting good practices in/ between CHCs is important.	4,44	4,71	3,78	4,10
32	Spreading and supporting good practices in the country/ between countries are important.	4,31	4,45	3,50	3,81
33	Coordination the care providers in the Community Health Centre is important.	4,41	4,65	3,75	4,00
34	Coordination between Community Health Centres (CHCs) (CHC = a model of care) is important.	4,16	4,35	3,50	3,77
35	Coordination between different region that provide health care is important.	4,03	4,19	3,25	3,52
	A total average value	4,27	4,43	3,70	3,94

Tables 5 and 6 present the mean, median, standard deviation, and minimum/maximum values from the final round.

**Table 5.** Acceptability of statements Table 5 long description.

ACCEPTABILITY OF STATEMENTS AVERAGE: average value of the first round	N	Mean	Median	Std. Deviat	Min	Max
[Community Health Centres respect fundamental human rights. AVERAGE: 4.50]	31	4.84	5	0.374	4	5
[Community Health Centres are strongly committed to being accessible for individuals and families, irrespective of race, religion, social status and other factors, including ability to pay for care. AVERAGE: 4.66]	31	4.84	5	0.454	3	5
[Collaboration between care providers in the Community Health Centre is important. AVERAGE: 4.56]	31	4.77	5	0.56	3	5
[Community Health Centres deliver integrated, comprehensive and people-centred primary health care. AVERAGE: 4.56]	31	4.74	5	0.445	4	5
[Spreading and supporting good practices in/ between CHCs is important. AVERAGE: 4.44]	31	4.71	5	0.529	3	5
[Community Health Centres have a commitment to equity and social inclusion. AVERAGE: 4.47]	31	4.68	5	0.475	4	5
[Community Health Centres engage in processes of continuous quality improvement. AVERAGE: 4.47]	31	4.68	5	0.541	3	5
[Clinical primary care services at Community Health Centres address aspects of health promotion and illness prevention. AVERAGE: 4.47]	31	4.65	5	0.608	3	5
[Coordination the care providers in the Community Health Centre is important. AVERAGE: 4.41]	31	4.65	5	0.608	3	5
[Community Health Centres deliver a care model through an interprofessional team. AVERAGE: 4.44]	31	4.61	5	0.558	3	5
[Community Health Centres contribute to universal coverage. AVERAGE: 4.34]	31	4.61	5	0.615	3	5
[Community Health Centres engage in needs of individuals and patients that they are serving. AVERAGE: 4.38]	31	4.61	5	0.558	3	5
[Community Health Centre is defined as a model of care in the specific region/ municipality/ district/ geographic area. AVERAGE: 4.31]	31	4.58	5	0.672	3	5
[Community Health Centres put emphasis on access to health care (with special attention given to the most vulnerable). AVERAGE: 4.38]	31	4.58	5	0.72	2	5
[Community Health Centres address aspects of both health and wellbeing. AVERAGE: 4.31]	31	4.52	5	0.626	3	5
[The Community Health Centre care team looks at the social determinants of health. AVERAGE: 4.41]	31	4.52	5	0.724	2	5
[Community Health Centres develop a community-oriented primary care strategy. AVERAGE: 4.31]	31	4.45	5	0.723	3	5
[Spreading and supporting good practices in the country/ between countries are important. AVERAGE: 4.31]	31	4.45	5	0.624	3	5
[Community Health Centres blend skills for individual health care with approaches focused on public health. AVERAGE: 4.25]	31	4.42	5	0.672	3	5
[Collaboration between Community Health Centres (CHCs) (CHC = a model of care) is important. AVERAGE: 4.22]	31	4.42	4	0.62	3	5
[Clinical primary care services at Community Health Centres use a holistic frame of reference. AVERAGE: 4.13]	31	4.35	4	0.709	3	5
[Clinical primary care services at Community Health Centres are orientated towards the needs of individuals. AVERAGE: 4.44]	31	4.35	5	0.798	2	5
[The Community Health Centre care team addresses social determinants of health through intersectoral cooperation. AVERAGE: 4.19]	31	4.35	4	0.755	2	5
[Community Health Centres take responsibility for a population that is geographically-determined. AVERAGE: 4.28]	31	4.35	4	0.608	3	5
[Coordination between Community Health Centres (CHCs) (CHC = a model of care) is important. AVERAGE: 4.16]	31	4.35	4	0.551	3	5
[Clinical primary care services at Community Health Centres are orientated towards the needs of communities. AVERAGE: 4,03]	31	4.32	4	0.702	3	5
[The Community Health Centre care team integrates into its daily activities, attention to the broader causes of illness. AVERAGE: 4.31]	31	4.32	4	0.599	3	5
[Clinical primary care services at Community Health Centres address aspects of curative care and rehabilitation. AVERAGE: 4.28]	31	4.29	4	0.824	2	5
[Collaboration between different region that provide health care is important. AVERAGE: 4.16]	31	4.26	4	0.631	3	5
[Community Health Centres place a strong emphasis on community engagement and civic participation in health and health care. AVERAGE: 4.00]	31	4.19	4	0.703	3	5
[Coordination between different region that provide health care is important. AVERAGE: 4.03]	31	4.19	4	0.703	2	5
[Clinical primary care services at Community Health Centres are orientated towards the needs of populations. AVERAGE: 3,91]	31	4.1	4	0.746	3	5
[Clinical primary care services at Community Health Centres are orientated towards the needs of families. AVERAGE: 4.00]	31	3.9	4	0.7	3	5
[Community Health Centres may include participation of clients/patients and other community members in governance of the healthcare organisation. AVERAGE: 3.78]	31	3.71	4	0.973	2	5
[Community Health Centres take responsibility for a population that is defined by population group(s). AVERAGE: 3.53]	31	3.61	4	0.989	2	5

**Table 6.** Feasibility of statements Table 6 long description.

FEASIBILITY OF STATEMENTS AVERAGE: average value of the first round	N	Mean	Median	Std. Deviat	Min	Max
[Community Health Centres are strongly committed to being accessible for individuals and families, irrespective of race, religion, social status and other factors, including ability to pay for care. AVERAGE: 4.28]	31	4.48	5	0.724	2	5
[Community Health Centres respect fundamental human rights. AVERAGE: 4.28]	31	4.42	4	0.62	3	5
[Clinical primary care services at Community Health Centres address aspects of health promotion and illness prevention. AVERAGE: 4.03]	31	4.35	4	0.755	2	5
[Clinical primary care services at Community Health Centres are orientated towards the needs of individuals. AVERAGE: 4.09]	31	4.29	4	0.739	3	5
[Community Health Centre is defined as a model of care in the specific region/ municipality/ district /geographic area. AVERAGE: 3.66]	31	4.23	4	0.762	3	5
[Community Health Centres deliver a care model through an interprofessional team. AVERAGE: 3.72]	31	4.23	4	0.884	2	5
[Community Health Centres deliver integrated, comprehensive and people-centered primary health care. AVERAGE: 3.88]	31	4.19	4	0.792	3	5
[Community Health Centres contribute to universal coverage. AVERAGE: 3.97]	31	4.19	4	0.946	1	5
[Community Health Centres put emphasis on access to health care (with special attention given to the most vulnerable). AVERAGE: 3.94]	31	4.16	4	0.735	2	5
[Community Health Centres engage in processes of continuous quality improvement. AVERAGE: 3.97]	31	4.16	4	0.86	1	5
[Clinical primary care services at Community Health Centres address aspects of curative care and rehabilitation. AVERAGE: 4.06]	31	4.13	4	0.991	1	5
[Community Health Centres engage in needs of individuals and patients that they are serving. AVERAGE: 3.91]	31	4.13	4	0.619	3	5
[Collaboration between care providers in the Community Health Centre is important. AVERAGE: 3.84]	31	4.1	4	0.79	2	5
[Spreading and supporting good practices in / between CHCs is important. AVERAGE: 3.78]	31	4.1	4	0.831	2	5
[Community Health Centres address aspects of both health and wellbeing. AVERAGE: 3.81]	31	4.03	4	0.795	2	5
[Coordination the care providers in the Community Health Centre is important. AVERAGE: 3.75]	31	4	4	0.632	3	5
[Clinical primary care services at Community Health Centres use a holistic frame of reference. AVERAGE: 3.66]	31	3.94	4	0.998	2	5
[The Community Health Centre care team looks at the social determinants of health. AVERAGE: 3.56]	31	3.94	4	0.998	1	5
[Community Health Centres take responsibility for a population that is geographically-determined. AVERAGE: 3.78]	31	3.9	4	1.012	1	5
[Collaboration between Community Health Centres (CHCs) (CHC = a model of care) is important. AVERAGE: 3.72]	31	3.84	4	0.86	2	5
[Spreading and supporting good practices in the country/ between countries are important. AVERAGE: 3.50]	31	3.81	4	1.014	2	5
[Clinical primary care services at Community Health Centres are orientated towards the needs of populations. AVERAGE: 3.53]	31	3.77	4	1.055	1	5
[Community Health Centres have a commitment to equity and social inclusion. AVERAGE: 3.75]	31	3.77	4	0.92	1	5
[Coordination between Community Health Centres (CHCs) (CHC = a model of care) is important. AVERAGE: 3.50]	31	3.77	4	0.884	1	5
[Clinical primary care services at Community Health Centres are orientated towards the needs of communities. AVERAGE: 3.47]	31	3.74	4	0.893	2	5
[The Community Health Centre care team addresses social determinants of health through intersectoral cooperation. AVERAGE: 3.38]	31	3.74	4	1.032	1	5
[Community Health Centres develop a community-oriented primary care strategy. AVERAGE: 3.38]	31	3.74	4	0.893	2	5
[The Community Health Centre care team integrates into its daily activities, attention to the broader causes of illness. AVERAGE: 3.56]	31	3.71	4	0.864	1	5
[Community Health Centres blend skills for individual health care with approaches focused on public health. AVERAGE: 3.59]	31	3.71	4	0.864	2	5
[Clinical primary care services at Community Health Centres are orientated towards the needs of families. AVERAGE: 3.59]	31	3.68	4	0.748	2	5
[Community Health Centres place a strong emphasis on community engagement and civic participation in health and health care. AVERAGE: 3.28]	31	3.68	4	0.945	1	5
[Collaboration between different region that provide health care is important. AVERAGE: 3.41]	31	3.65	4	0.877	2	5
[Community Health Centres take responsibility for a population that is defined by population group(s). AVERAGE: 3.34]	31	3.61	4	1.054	1	5
[Coordination between different region that provide health care is important. AVERAGE: 3.25]	31	3.52	4	0.926	2	5
[Community Health Centres may include participation of clients/patients and other community members in governance of the healthcare organisation. AVERAGE: 3.22]	31	3.29	3	1.039	1	5

All statements were deemed both acceptable and feasible by participants. Statements assessed in the second round were generally rated slightly higher than in the first round. Feasibility scores were consistently somewhat lower than acceptability scores. Across all statements, the overall mean score was 4.43 for acceptability and 3.94 for feasibility, with a median of 4 or 5 for both dimensions, except for the governance-related statement noted above, which had a median feasibility score of 3. The data show greater variability in feasibility ratings (minimum 1; maximum 5; SD ≈0.87) compared with acceptability ratings (minimum 1; maximum 5; SD ≈0.65).

### Acceptability of the Community Health Centres (CHCs) as a model of care

The analysis identified a cluster of statements with very high levels of agreement (Mean >4.5). These included respect for fundamental human rights (Mean = 4.84; SD = 0.374), accessibility irrespective of socio economic or demographic characteristics (Mean = 4.84; SD = 0.454), collaboration between care providers (Mean = 4.77; SD = 0.560) and the delivery of integrated, comprehensive and people centred PHC (Mean = 4.74; SD = 0.445). Additional highly rated items included equity and social inclusion (Mean = 4.68; SD = 0.475), continuous quality improvement (Mean = 4.68; SD = 0.541) and an emphasis on prevention and health promotion (Mean = 4.65; SD = 0.608). These statements were characterized by relatively low standard deviations (SD ≈0.374–0.615), indicating a high degree of consensus among respondents.

Participants also emphasized the importance of collaboration and coordination among care providers within and between CHCs, as well as the dissemination and support of best practices. Mean scores for these statements ranged from 4.61 to 4.84, with SD values between approximately 0.37 and 0.62.

In contrast, the lowest rated statements included responsibility defined by population groups (Mean = 3.61; SD = 0.989), participation of patients and community members in governance (Mean = 3.71; SD = 0.973) and orientation towards families (Mean = 3.90; SD = 0.700). These items exhibited both lower mean values and notably higher standard deviations (SD ≈0.70–0.99), suggesting greater variability in respondents’ views.

### Feasibility of the Community Health Centres (CHCs) as a model of care

The findings indicate that several core components of the CHC model are perceived as highly feasible, with mean values above 4.0. These include accessibility regardless of socio-economic or demographic characteristics (Mean = 4.48; SD = 0.724), respect for fundamental human rights (Mean = 4.42; SD = 0.620), the provision of preventive and promotive care (Mean = 4.35; SD = 0.755) and an orientation towards individual patient needs (Mean = 4.29; SD = 0.739). Relatively high feasibility scores were also observed for integrated and people-centred care (Mean = 4.19; SD = 0.792), interprofessional teamwork (Mean = 4.23; SD = 0.884) and contributions to universal health coverage (Mean = 4.19; SD = 0.946).

In contrast, the lowest-rated statements (Mean = 3.29–3.52) related primarily to governance, participation and coordination beyond the individual CHC level. These included participation of patients and community members in governance (Mean = 3.29; SD = 1.039), coordination between regions (Mean = 3.52; SD = 0.926), defining responsibility based on population groups (Mean = 3.61; SD = 1.054) and community engagement and civic participation (Mean = 3.68; SD = 0.945). These items were characterized by both lower mean values and higher standard deviations, indicating greater variability in respondents’ views.

Consistent with the acceptability ratings, the highest feasibility scores (Mean = 4.00–4.48; SD ≈0.62–0.99) corresponded to statements suggesting that the CHC model is particularly effective when implemented within clearly defined geographical areas.

Overall, the CHC model that emerged from the study was supported across all 35 statements, with consensus achieved in both rounds of the Delphi process (Table 4), although perceptions of feasibility showed somewhat greater variability. In addition to the existing IFCHC definition, the resulting model places particular emphasis on cooperation and coordination within and between health centres at the regional level, as well as on the dissemination and exchange of knowledge, representing a newly introduced and distinctive component of the European CHC framework.

## Discussion

### Methods

The IFCHC definition encompasses the main groups of activities undertaken by CHCs. However, this study sought to determine whether additional elements were considered important by experts across Europe. Discussions during the workshops highlighted collaboration and the dissemination of good practices as additional dimensions that warranted inclusion in the Delphi questionnaire. Although the workshop-based data collection resembled an expert consensus process, it was not formally classified as such due to the informal nature of the workshop settings. Once the list of 35 statements had been finalized, a two-round Delphi process was selected as the most appropriate qualitative method for assessing agreement. Participants were not able to add new statements or provide open comments, as this stage of content generation had already been completed during the workshops.

The Delphi study facilitated cross-national participation among individuals who did not know one another, while allowing time for reflection on the proposed statements. The observed increase in agreement from the first to the second round for most items suggests that opportunities for reconsideration can foster deeper understanding and strengthen consensus, an established advantage of the Delphi method.

Because participants did not know the study coordinator personally, the invitation to participate may have been perceived as impersonal, which may partly explain the modest response rate. The extended timeline and the need for repeated follow-up reminders also contributed to the prolonged duration of the study. Importantly, the second-round questionnaire was sent only to those who completed the first round, all of whom eventually responded after repeated reminders. Purposive sampling of individuals with substantial expertise in primary care enabled context-specific assessment relevant to diverse national settings. This approach did not introduce bias, as participants provided their views anonymously and without knowledge of one another’s identities. All participants had considerable experience in the field, and diversity was ensured by recruiting experts from multiple sectors of PHC (Nasa *et al*., 2021; Naisola Ruiter, 2022; Shang *et al*., 2023).

Consensus is a central requirement of Delphi studies, although the concept itself is often criticized for lacking a universally accepted definition. Consensus thresholds vary widely in the literature, ranging from 51% to 100%, and are frequently selected arbitrarily (Barrett *et al*., 2020; Nasa *et al*., 2021). In this study, the agreement threshold was predefined as part of the methodological design and was successfully achieved.

### Participants

The 16 participating countries represented a wide spectrum of primary care contexts, including those with strong primary care systems (e.g. Belgium, Denmark, Finland, the Netherlands, Portugal, Slovenia, Spain, the UK), as identified in the PHAMEU study (Kringos *et al*., 2013), as well as countries with more moderate or developing primary care structures (e.g. Italy, Portugal) and those with comparatively lower investment in primary care (e.g. North Macedonia, Croatia, Turkey). This diversity also reflected variation in health system organization, including both Beveridge- and Bismarck-type insurance models, thereby ensuring a broad range of perspectives and experiences. Participants worked in a variety of professional environments, including CHCs, group practices, academic institutions, professional organizations and public agencies. This diversity enabled the inclusion of multiple viewpoints on the functioning and organization of primary care. Representation from both urban and rural settings added an additional layer of insight, particularly regarding differences in service delivery, accessibility and community engagement. Panel members were current or former EFPC affiliates with recognized expertise in primary care, selected to strengthen the qualitative robustness of the consensus process.

### Primary health care models versus the Community Health Centre as a model of care

PHC provides integrated, holistic, community- and patient-centred care for populations (Binagwaho *et al*., 2019; WHO, 2022). It is important to distinguish between the content of care and the model through which it is delivered. Many of the highly rated elements in this study, such as accessibility, equity, holistic care, interprofessional collaboration and attention to social determinants of health, are widely recognized principles of high-quality primary care and are not unique to CHCs in terms of the services they provide. The added value of CHCs lies in the way these principles are operationalized. CHCs represent an organizational model in which these elements are systematically implemented, integrated and institutionalized through structured interprofessional teamwork, alignment with social services, population-based responsibility and community engagement (De Maeseneer *et al*., 2019). The effectiveness of primary care depends not only on its core functions but also on how consistently these functions are organized and delivered. Thus, the distinctiveness of CHCs does not lie in the principles themselves, but in their integration within a coherent organizational structure.

This aligns with the understanding of PHC as a system-level approach, which is much easier to deliver within CHCs than as a set of discrete services (Nagel *et al*., 2021). This interpretation is consistent with the system-level perspective demonstrated in the PHAMEU research led by Kringos *et al*. (2013), which identified substantial variation in the strength of primary care across European countries. Countries with strong primary care systems tend to have more developed organizational structures that support integrated, community oriented care, making them particularly conducive environments for CHC type models. Furthermore, the 2025 report of the Canadian Association of CHCs (The Canadian Association of Community Health Centres, 2025) reaffirms similar conclusions, emphasizing that CHCs are most effective when embedded within strong primary care systems and supported by policies that promote integration, equity and community participation. These findings reinforce the relevance of the CHC model as a structured approach to delivering comprehensive primary care.

### Contextualizing the CHC model of care

The CHC model was defined through 35 key statements, of which 32 and 16 received average acceptability and feasibility scores of ≥4 on a 5-point Likert scale (4 = agree, 5 = strongly agree), respectively. This reflects strong endorsement of CHCs as interprofessional, team based primary care structures integrated with broader health, social and community services, consistent with findings from previous research (Nagel *et al*., 2021; Saloner *et al*., 2020). All remaining statements also had average values above 3.2, indicating general agreement in principle. The median values show that most respondents provided high ratings, while the standard deviations indicate some dispersion, suggesting that although opinions were not entirely unanimous, the overall trend was strongly positive. It is understandable that agreement with statements defining health care as a model of care is more relevant to acceptability than to feasibility in practice.

Although differences across work settings, countries or participant groups might have been informative, such comparisons were not undertaken. The study aimed to develop a uniform template for CHC implementation across Europe, and the focus therefore remained on achieving consensus among all participants.

Core elements such as health promotion, community engagement and responsibility for a defined population were considered both appropriate and essential for contemporary European health systems. These values align with global policy frameworks on primary care, including the Alma-Ata and Astana Declarations (Chan *et al*., 2008; Kluge *et al*., 2019), and reflect WHO Europe’s vision for integrated and equitable care (World Health Organisation, 2022).

Although the model’s acceptability was overwhelmingly high, participants expressed greater caution regarding its feasibility, particularly in components requiring complex organizational or governance structures. This likely reflects their direct experience with the constraints and variability inherent in their respective health systems.

The social mission of CHCs, grounded in respect for human rights, accessibility and inclusion, was strongly affirmed. Statements emphasizing universal access and integrated, people-centred care received the highest scores, reinforcing earlier findings that CHCs are well-positioned to promote health equity (De Maeseneer *et al*., 2019; Pourat *et al*., 2023). This consensus highlights CHCs as fundamentally inclusive and ethically grounded institutions.

Statements relating to social determinants of health and community engagement received high acceptability but slightly lower feasibility scores, indicating broad agreement with the principles but recognition of the practical challenges associated with implementation.

An important theme emerging from workshops, though not explicitly included in the IFCHC definition, was the role of CHCs in disseminating good practice, fostering inter-regional learning, and building shared knowledge bases. European networks such as WONCA, EFPC and EQUIP actively support these processes through conferences, communities of practice and cross-border collaboration (EFPC, 2020).

High scores for interprofessional collaboration and coordination among care providers underscored the value of team-based care within CHCs. Evidence links effective collaboration to improved outcomes and patient satisfaction. However, this is not yet standard practice in all settings, and further investment in interprofessional education is needed. Such education supports professionals in developing collaborative competencies and complements mono-professional training by fostering confidence and mutual understanding (Miller *et al*., 2019).

Given ongoing demographic shifts, migration and increasing cultural diversity, coordination across regions and providers is essential for delivering consistent, culturally sensitive, high-quality care tailored to local populations. Research supports the benefits of cross-regional and cross-national cooperation, especially in responding to migration and refugee health needs (Lebano *et al*., 2020; Priebe *et al*., 2011).

Familycentred care, one of the lowestrated items, depends simultaneously on provider skills, family dynamics and organizational support. Panellists likely perceived low feasibility because these models require systemlevel redesign rather than incremental practice change, as well as the capacity to manage vulnerable or specific population groups, an approach widely recommended but difficult to implement. Barriers include socioeconomic disadvantage, low health literacy and weak social support systems. Programmes often fail when services do not match community needs. These interventions require tailored, contextsensitive approaches that are resourceintensive and difficult to standardize, which may explain the lower feasibility ratings. Both familycentred care and targeted population approaches are conceptually strong but operationally demanding, which helps explain why experts consistently judged them challenging to implement in realworld primary care (Pourat *et al*., 2023). These lower scores likely reflect structural, legislative or cultural barriers, as well as uncertainty or skepticism about practical implementation, highlighting areas for further education and innovation.

The lowest score was assigned to community involvement in governance. While in some jurisdictions the mandate and direction of CHCs may be determined solely by the state, in many contexts, policy explicitly requires that boards of directors include active participation from community stakeholders. Strong leadership and effective management are therefore essential for maintaining relationships with the community, fostering collaborative partnerships and ensuring the efficient day-to-day operation (Nagel *et al*., 2021). The current definition of CHCs as a model of care did not sufficiently emphasize the role of management, despite its critical importance in fostering staff engagement and service quality (Szilvassy *et al*., 2022). This omission may reflect participants’ focus on the substantive characteristics of the CHC model rather than operational considerations. Nonetheless, effective management significantly influences employee performance and care delivery (Curry *et al*., 2020). Despite these concerns, the literature supports the potential of participatory governance and distributive leadership to enhance transparency, trust and responsiveness.

For example, CHCs in the Netherlands incorporate client advisory boards or cooperative ownership models that enable community influence (Schäfer *et al*., 2010). Swedish regional governance often includes citizen representatives on health committees (Saltman *et al*., 2007). Italy’s Tuscany region has experimented with patientrepresentative health councils (Nuti *et al*., 2012), while in Flanders, patients and informal caregivers participate in Boards of Directors within Primary Care Zones (World Health Organisation, 2019).

Conversely, governance in Central and Eastern Europe (e.g. Poland, Slovenia) tends to remain top-down and professionally driven, with limited civic participation (Kowalska-Bobko *et al*., 2020; Zavrnik *et al*., 2021). These systemic contrasts help explain feasibility differences and underscore the need for adaptable frameworks that respect national contexts while gradually integrating participatory elements.

Community-based care and co-production in service design offer additional promise. Involving patients and community members in identifying priorities and developing interventions enhances service relevance, satisfaction and outcomes (Morton *et al*., 2020; Rijken *et al*., 2021). Migrant-sensitive and culturally adapted pathways are essential for improving quality and equity, particularly in diverse urban settings (Bradby *et al*., 2015; Ingleby *et al*., 2012).

The experience of the EFPC demonstrates the power of global collaboration among professionals across disciplines, including those in the social sector, as a vital strategy to strengthening primary care in Europe and beyond (De Maeseneer *et al*., 2017).

### CHC – A feasible European model of care

The IFCHC definition, expanded in this study through additional statements on collaboration and knowledge dissemination, was fully endorsed by the panellists, who viewed CHCs as a concept aligned with a European model of care. The shift from conceptual acceptance to practical implementation indicates that, while the model is normatively supported, its operationalization remains less certain. This variability may be linked to differences in governance and financing arrangements, workforce capacity, health system models and levels of coordination across settings, all of which may influence the feasibility of implementation (European Observatory on Health Systems and Policies, 2015).

Nevertheless, CHCs as a model for delivering health care uphold fundamental principles of PHC. In the context of rapidly evolving health system demands, it is essential that population-based care delivered through the CHC model remains timely, comprehensive and responsive to people’s needs. This can be achieved more effectively when cooperation, coordination and the exchange of knowledge and experience are explicitly emphasized, as highlighted by the findings of this study.

## Conclusion

The European CHCs model of care provides both preventive and curative services to all individuals within a defined geographical area. Care is delivered equitably and with full respect for human rights and social identity, through interprofessional teams grounded in integrated, comprehensive and peoplecentred PHC.

While CHCs must cultivate a community-oriented primary care strategy and maintain a strong public health focus, their foremost priority is to respond to the needs of individuals, followed by those of families and the wider community. Health care providers working within CHCs should promote cooperation at local, regional, national and international levels to support the exchange of knowledge and best practices. The model therefore emphasizes not only the nature of care delivered but also the collaborative ethos through which it is organized.

The findings demonstrate strong ideological endorsement of the CHC model, particularly its inclusive orientation, team-based structure and holistic philosophy of care. Although feasibility currently lags slightly behind acceptability, the upward trend suggests increasing optimism regarding realworld implementation. Governance, interprofessional collaboration and intersectoral coordination emerge as areas requiring particular attention, as feasibility in these domains remains more variable.

Strengthening and formalizing CHCs’ commitment to fundamental human rights, accessibility, integrated and peoplecentred care and intrateam collaboration clearly represent top priorities, enjoying near-universal consensus. Operationalizing these principles, across governance structures, funding mechanisms, workforce policies and care pathways, holds the greatest potential for strategic and policy impact. Conversely, elements such as client and patient participation in governance and population-level responsibility for defined groups, while broadly acceptable, appear less central within the current framework.

## Data Availability

The data supporting the findings of this study are available upon request from the corresponding author.

## Table 1. Long description

A table defining the characteristics and principles of Community Health Centres with 35 statements. The table outlines the key components of the definition developed by the International Federation of Community Health Centres (IFCHC). It includes statements about the model of care, integrated and comprehensive primary health care, interprofessional teams, health and wellbeing, health promotion, illness prevention, curative care, rehabilitation, holistic frame of reference, individual and family needs, social determinants of health, community-oriented primary care strategy, equity, social inclusion, access to health care, human rights, community engagement, civic participation, universal coverage, accessibility, continuous quality improvement, population responsibility, collaboration, and coordination. The table has 35 rows and 1 column, each row representing a distinct statement.

Navigate back to Table 1..

## Table 2. Long description

The table presents data on participants from 16 European countries. It includes three columns: Country, Number, and Percent. The countries listed are Belgium, Bosnia and Herzegovina, Croatia, Denmark, Estonia, France, Germany, Italy, Macedonia, Malta, Montenegro, Netherlands, Portugal, Slovenia, Spain, and Turkey. Each country has a corresponding number of participants and their percentage of the total. Belgium, Germany, Netherlands, Slovenia, and Turkey each have 3 participants, accounting for 9.7 percent each. Croatia, Italy, Malta, Portugal, and Spain each have 2 participants, accounting for 6.5 percent each. Bosnia and Herzegovina, Denmark, Estonia, France, Macedonia, and Montenegro each have 1 participant, accounting for 3.2 percent each. The total number of participants is 31, making up 100 percent.

Navigate back to Table 2..

## Table 3. Long description

The table presents data on the working settings of participants, categorized by type, number, and percentage. It includes six rows and three columns. The columns are labeled ‘Working IN’, ‘N’, and ‘Percent’. The rows list different working settings: Group practice, Community Health Centre, Others (e.g., university), Professional organisation, and State institution, along with a total row. The ‘N’ column shows the number of participants in each setting, while the ‘Percent’ column shows the corresponding percentage. Notable trends include the highest percentage of participants working in Community Health Centres at 41.9 percent, followed by State institutions at 19.4 percent, and Group practice at 9.7 percent. The ‘Others’ category accounts for 16.1 percent, and Professional organisations account for 12.9 percent. The total number of participants is 31, making up 100 percent.

Navigate back to Table 3..

## Table 4. Long description

The table presents a comparison of the first-round results and the second-round results for both acceptability and feasibility of the Community Health Centres (CHC) model. It includes 35 statements evaluated by participants. Each statement is scored on a scale, with the first round showing individual agreement and the second round showing consensus among participants. The table lists the mean scores for acceptability and feasibility for each statement in both rounds. Notable trends include the achievement of consensus on all statements after the second round, with mean scores reaching at least 3. The lowest mean score for acceptability was 3.61, and for feasibility, it was 3.52, except for one statement which received a feasibility score of 3.29. The table has 35 rows and 6 columns, with column headers indicating the statement numbers, definitions, and scores for acceptability and feasibility in both rounds.

Navigate back to Table 4..

## Table 5. Long description

The table presents data on the acceptability of various statements related to Community Health Centres. It includes columns for the statement descriptions, number of respondents, mean, median, standard deviation, minimum, and maximum values. The statements cover aspects such as respect for human rights, accessibility, collaboration, good practices, equity, social inclusion, quality improvement, health promotion, and prevention. Each statement is evaluated based on responses from 31 participants, with mean values ranging from 3.31 to 4.84, medians ranging from 3 to 5, and standard deviations indicating variability in responses.

Navigate back to Table 5..

## Table 6. Long description

The table presents the feasibility of various statements related to Community Health Centres, showing the average values from the first round of a survey. It includes columns for the number of responses, mean, median, standard deviation, minimum, and maximum values. The rows list different statements about the commitment, respect for human rights, health promotion, and other aspects of Community Health Centres. Notable trends include high mean values for statements about accessibility, respect for human rights, and health promotion, indicating strong agreement among respondents. The table provides a detailed overview of the survey results, highlighting areas of consensus and variation.

Navigate back to Table 6..
